# Assembly of the *Delphinium densiflorum* Chloroplast Genome and Comparative Genomics Within *Delphinium*

**DOI:** 10.3390/genes17020240

**Published:** 2026-02-17

**Authors:** Siqi Chen, Min Wang, Xinhang Lu, Yuying Sun, Min Ma

**Affiliations:** 1 Qinghai Engineering Research Center of Modern Tibetan Medicine Development, Key Laboratory for Tibet Plateau Phytochemistry of Qinghai Province, School of Pharmacy, Qinghai Minzu University, Xining 810007, China; chensiqi0809@163.com (S.C.); 13619780817@163.com (M.W.); 2 School of Modern Plateau Biomedical Industry, Qinghai Minzu University, Xining 810007, China; 3 College of Ecological Environment and Resources, Qinghai Minzu University, Xining 810007, China; xiaoxiao59408@163.com (X.L.); syy19963964692@outlook.com (Y.S.)

**Keywords:** *Delphinium densiflorum*, chloroplast genome, comparative genomics

## Abstract

**Background/Objectives**: Chloroplast genomes are essential for understanding the systematics and adaptive evolution of alpine plants, yet genomic data for high-altitude *Delphinium* species remain scarce. *Delphinium densiflorum*, a medicinal plant endemic to the Qinghai-Tibet Plateau, exhibits notable high-altitude adaptations, but its plastome features and evolutionary position are still unclear. This study aims to assemble and characterize its complete chloroplast genome and clarify its phylogenetic placement within *Delphinium*. **Methods**: Using Illumina NovaSeq data, we de novo assembled the *D. densiflorum* plastome, annotated it with CPGAVAS2, and compared it with 12 published Ranunculaceae plastomes. We analyzed IR-boundary dynamics, genome-wide sequence variation, and codon-usage bias and constructed a maximum-likelihood phylogeny based on 69 shared protein-coding genes. **Results**: The plastome is 154,161 bp (GC 38.24%) with a canonical quadripartite structure, encoding 131 genes (87 CDS, 8 rRNA, 37 tRNA). An IR expansion into the SSC region yields the shortest SSC reported among the compared *Delphinium* species and produces unique structural variants. Photosynthetic genes are extremely conserved (nucleotide diversity Pi ≤ 0.01), whereas several loci (e.g., *ycf1* and *psaC*) are highly divergent (Pi ≥ 0.05). Codon usage shows a strong bias toward AU-ending triplets. Phylogenetically, *D. densiflorum* forms a 100%-bootstrap clade with other high-altitude congeners, supporting the non-monophyly of *Delphinium*. **Conclusions**: This study delineates the plastome architecture and putative adaptive signatures of *D. densiflorum*, identifies robust candidate loci for DNA barcoding, and provides molecular evidence for taxonomic revision and conservation strategies in *Delphinium*.

## 1. Introduction

*Delphinium densiflorum* is a perennial herb endemic to the Qinghai-Tibet Plateau and adjacent highlands, where it occurs in valley thickets, river terraces, or alluvial fans at elevations of 3300–4500 m. The entire plant is used in traditional Tibetan and folk medicine as an antipyretic and detoxifying agent; decoctions are applied externally to treat dermatitis, furuncles, tinea, and psoriasis or taken orally to counteract Aconitum poisoning [1].

The genus *Delphinium* L. (Ranunculaceae) comprises approximately 365 species distributed throughout the north-temperate alpine and sub-alpine belts, with three primary diversity centres: (i) the European Alps-Carpathians, (ii) the Rocky Mountains-Mexican Plateau, and (iii) the Himalaya-Hengduan Mountains [2,3]. China harbours the richest assemblage, with 232 species (≈64% of global diversity) recorded to date [4].

For centuries, the tubers and roots of *Delphinium* have been employed in traditional materia medica across continents. In Anatolian folk medicine, they are prescribed against epilepsy, rabies, and tetanus [5]; in the UK and France, the dried and powdered tubers of *D. staphisagria* and *D. peregrinum* are used as an “insecticidal powder”; in military camps it is dusted onto clothing to kill body lice, and the same formula is also applied externally for scabies and cutaneous parasitic infections [3]; in Iran they are administered for splenic disorders, jaundice, and oedema [6], whereas in China preparations are utilised to relieve toothache, rheumatic pain, oedema, and cutaneous affections such as scabies and tinea [7].

Orogenic uplift of the Qinghai-Tibet Plateau since the Late Miocene, coupled with intensified monsoon dynamics and Quaternary climatic oscillations, has triggered rapid radiation, recurrent hybridisation, and extensive polyploidisation within *Delphinium*, producing widespread morphological convergence and cytotype mosaics that confound both classical taxonomy and molecular phylogenetic reconstruction [8,9]. Concomitantly, the chloroplast (cp) genomes of high-alpine lineages have experienced unique molecular trajectories—ranging from gene loss and pseudogenisation to IR boundary shifts and accelerated sequence divergence—which have been hypothesized to reflect potential adaptation to hypobaric hypoxia, high UV-B flux, and thermal instability [10,11], although neutral processes such as genetic drift and mutational bias may additionally contribute. Despite these insights, the phylogenetic placement of *Delphinium* within Ranunculaceae remains contentious, with morphological circumscriptions often contradicting molecular topologies [8,12].

Chloroplasts retain a semi-autonomous genetic system that integrates environmental cues with organellar gene expression [13,14]. The circular cp genome of photosynthetic angiosperms (115–165 kb) is present in high copy number and exhibits strong structural conservatism, making it an ideal locus for comparative evolutionary studies [15]. Elucidating the complete cp genome of *D. densiflorum* will therefore (i) clarify the deep-level relationships between *Delphinium* and other ranunculaceous genera—especially *Ranunculus*, with which it shares superficial morphological similarity—and (ii) provide a robust reference for future DNA-barcode design, germplasm conservation, and marker-assisted breeding programmes within the family.

Here, we used Illumina NovaSeq paired-end sequencing to generate a high-quality, fully annotated chloroplast genome of *D. densiflorum*. Through comparative genomic and phylogenomic analyses that incorporate all publicly available ranunculaceous cp genomes, we aim to (i) characterise structural innovations unique to the high-alpine *Delphinium* lineage, (ii) test the monophyly of traditional infrageneric classifications, and (iii) refine the temporal and biogeographic framework of Ranunculaceae diversification.

## 2. Materials and Methods

### 2.1. Sample Collection and DNA Extraction

*D. densiflorum* samples were collected from Qunjia Forest Farm, Huangzhong District, Xining City, Qinghai Province, China (101°41′ E, 36°16′ N). Healthy fresh leaves were harvested, silica-dried, and transported back to the laboratory for subsequent use. Species identification was verified by Associate Professor Jiuli Wang from Qinghai Minzu University.

Genomic DNA was extracted using the Plant Genomic DNA Kit (Cat. No. DP305; Tiangen Biotech (Beijing) Co., Ltd., Beijing, China) strictly following the manufacturer’s protocols. The extracted DNA samples were then sent to Nanjing GSH Biotechnology Co., Ltd. (Nanjing, China) for high-throughput sequencing.

### 2.2. Sequencing and Data Quality Control

Following qualification of the genomic DNA samples, the DNA was fragmented by ultrasonication. The fragmented DNA was then subjected to purification, end repair, 3′-dA tailing, and sequencing adapter ligation. Fragment size selection was performed via agarose gel electrophoresis, followed by PCR amplification to construct the sequencing library. The constructed library underwent quality inspection; qualified libraries were sequenced on the Illumina NovaSeq 6000 platform with a paired-end (PE) read length of 150 bp (PE150) [16].

Raw sequencing data were filtered using fastp software (version 0.20.0, https://github.com/OpenGene/fastp, accessed on 13 January 2026): potential sequencing adapters and primer sequences were trimmed from the reads; reads with an average quality score < Q5 were discarded to avoid interference with subsequent analysis accuracy; reads containing more than 5 Ns (undetermined bases) were removed. Strict quality control was implemented to reduce uncertainties in the sequencing results [17].

### 2.3. Genome Assembly

The chloroplast genome of *D. densiflorum* was assembled using SPAdes software (v3.10.1, https://github.com/ablab/spades, accessed on 13 January 2026) with k-mer values set to 55, 87, and 121, yielding a preliminary chloroplast genome scaffold. Gap filling was performed on the assembled sequence using Gapfiller v2.1.1 (https://sourceforge.net/projects/gapfiller/, accessed on 13 January 2026) [18]. Based on the typical quadripartite structure of chloroplast genomes, the corrected pseudo-genome was subjected to coordinate rearrangement to generate a complete circular chloroplast genome sequence [19]. Finally, the final chloroplast genome sequence of *D. densiflorum* was obtained after manual inspection and refinement.

To assess the quality and completeness of the assembled chloroplast genome, the filtered clean reads were mapped back to the final assembly using BWA (0.7.19) (Burrows-Wheeler Aligner) [20] with default parameters. The mapping results were processed using SAMtools (v2.1) [21] to calculate genome coverage depth and breadth. The assembly achieved >500× mean coverage depth and 100% breadth (complete coverage of the chloroplast genome), with >99% of bases covered by at least 10 reads and >95% covered by at least 50 reads, confirming high completeness and reliability of the assembly.

### 2.4. Genome Annotation

The assembled complete chloroplast genome sequence was annotated using CPGAVAS2 (v2.1.0), a specialized tool for chloroplast genome annotation. Following manual inspection and correction, the chloroplast genome map was generated using Chloroplot (https://irscope.shinyapps.io/Chloroplot/, accessed on 13 January 2026), an organellar genome visualization tool. Finally, the annotated chloroplast genome was submitted to the GenBank database of the National Center for Biotechnology Information (NCBI) with the accession number OM022261.1.

### 2.5. Comparative Genomic Analysis

*D. densiflorum* belongs to the genus *Delphinium*. For comparative chloroplast genomic analysis, we selected 12 representative species with publicly available complete chloroplast genomes, including 11 *Delphinium* species (i.e., *Delphinium anthriscifolium*, *D. brunonianum* [22], *D. caeruleum*, *D. candelabrum* var. *monanthum*, *D. ceratophorum*, *D. denudatum* [23], *D. grandiflorum* [24], *D. maackianum*, *D. tangkulaense*, and *D. yunnanense*) and one Ranunculaceae outgroup species (*Ranunculus tanguticus*). Combined with the chloroplast genome of *D. densiflorum* obtained in this study, a total of 12 complete chloroplast genomes were included in the comparative analysis.

#### 2.5.1. IR Boundary Analysis

Previous studies have shown that the chloroplast genomes of *Delphinium* species typically possess a circular quadripartite structure, with four junction sites between the inverted repeat (IR) regions and single-copy (SC) regions: JSA (SSC-IRa), JLA (IRa-LSC), JLB (LSC-IRb), and JSB (IRb-SSC) [7]. IRscope (https://irscope.shinyapps.io/irapp/, accessed on 13 January 2026) was used to analyze the boundary positions between IR and SC regions of *D. densiflorum* and 11 other Ranunculaceae species, thereby determining the expansion and contraction events of the IR regions [25].

#### 2.5.2. Global Sequence Variation Analysis

The CPJSdraw visualization tool (http://cloud.genepioneer.com:9929, accessed on 13 January 2026) [26] was employed to compare and analyze the boundary information of the four regions (LSC, SSC, IRa, IRb) in the chloroplast genomes of 12 Ranunculaceae species, allowing observation of the contraction and expansion dynamics of the IR regions. Using the chloroplast genome of *D. densiflorum* as the reference, homologous sequence alignment was performed via the online mVISTA tool (https://genome.lbl.gov/vista/index.shtml, accessed on 13 January 2026) [27] under the Shuffle-LAGAN (global alignment) mode.

#### 2.5.3. Nucleotide Diversity Analysis

Nucleotide diversity (Pi) was calculated for protein-coding sequences (CDSs) and intergenic spacers (IGSs) using DnaSP v6.0 [28]. Complete chloroplast genome sequences of 12 species were aligned with MAFFT v7.0 [29] using the G-INS-i strategy. For CDS analysis, start and stop codons were excluded, and overlapping regions between adjacent genes were trimmed to avoid redundancy. For IGS analysis, intergenic regions between protein-coding and tRNA genes were extracted and concatenated. Pi values were computed using a sliding window approach (window size: 600 bp; step size: 200 bp) implemented in DnaSP to visualize the distribution of genetic diversity across the genome. Hypervariable hotspots were operationally defined as regions with Pi ≥ 0.06 for IGSs and Pi ≥ 0.02 for CDsS, whereas conserved loci were defined as Pi ≤ 0.01. These thresholds were selected based on the overall distribution of nucleotide diversity in the dataset.

### 2.6. Phylogenetic Tree Construction

In this study, *R. tanguticus* (GenBank ID: OR625580.1) was selected as the outgroup. Shared protein-coding sequences (CDSs) were extracted from the chloroplast genomes of *D. densiflorum* and its related taxa (Table 1). After sequence integration and alignment using MAFFT, the best-fit nucleotide substitution model was determined using ModelTest-NG (v0.1.7), which selected the GTR + I + G model as optimal based on the Akaike Information Criterion (AIC). A phylogenetic tree was constructed via MEGA X software (v10.0) based on the maximum likelihood (ML) method [30]. The main parameters were set as follows: Model = GTR + I + G, bootstrap = 1000 replicates. Bootstrap support values ≥ 70% were considered as strong support.

## 3. Results

### 3.1. Chloroplast Genome Characteristics

**Figure 1:**
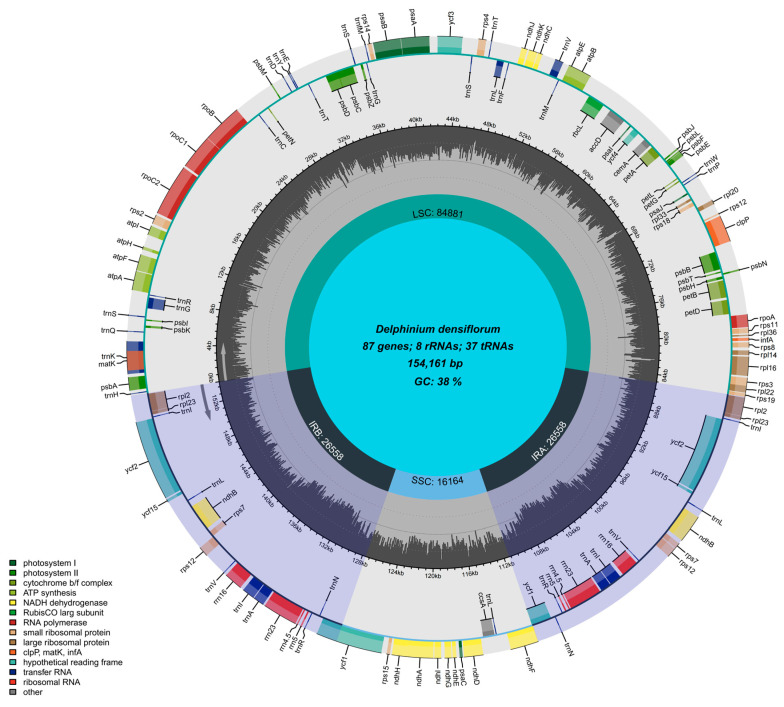
Chloroplast Genome Map of *D. densiflorum*. Genes drawn outside the circle are transcribed in the clockwise direction, whereas those inside are transcribed counter-clockwise. Different colored boxes denote distinct functional gene categories. The inner gray ring represents GC content, with darker peaks indicating regions of higher GC content. LSC, large single-copy region (green); SSC, small single-copy region (light blue); IRa and IRb, inverted repeat regions (light purple). Genome size: 154,161 bp; average GC content: 38%.

Approximately 5.01 Gb of paired-end sequencing data was generated in this study, providing an estimated coverage depth of ~507× (based on genome size of 154,161 bp) and 100% breadth, ensuring robust assembly quality. Read mapping validation showed that 99.7% of the chloroplast genome was covered by ≥10 reads and 96.2% by ≥50 reads, with an average mapping quality score of Q58, confirming high assembly accuracy and completeness. From these data, a circular chloroplast genome of *D. densiflorum* (Figure 1) was assembled, with a total length of 154,161 bp and a GC content of 38.24%. It exhibits a typical quadripartite structure consisting of a large single-copy (LSC) region of 84,881 bp, a small single-copy (SSC) region of 16,164 bp, and two identical inverted repeat (IR) regions (IRa and IRb) each of 26,558 bp.

The chloroplast genome of *D. densiflorum* contains 131 genes, which can be functionally classified into categories such as photosynthesis and self-replication (Table 2). Among these, there are 87 protein-coding sequences (CDSs), 8 ribosomal RNA (rRNA) genes, and 37 transfer RNA (tRNA) genes, with no pseudogenes detected.

### 3.2. IR Boundary Analysis Among Delphinium Species

**Figure 2:**
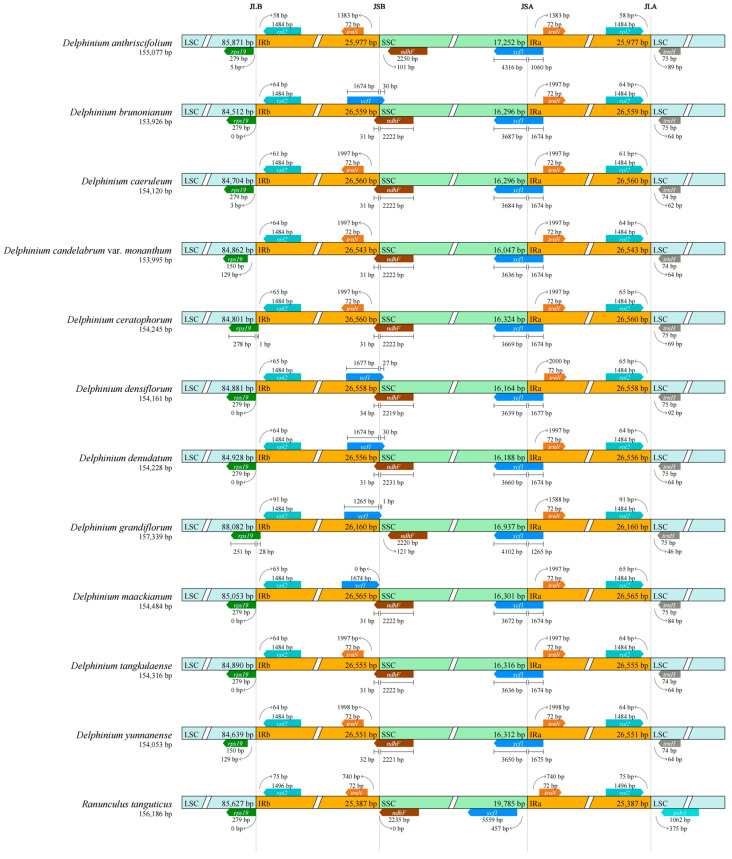
Comparative analysis of the four junction sites (JLB, JSB, JSA, and JLA) between the inverted repeat (IRa, IRb) and single-copy (LSC, SSC) regions in twelve Delphinium and one Ranunculus chloroplast genomes. Genes adjacent to the junctions are shown, with numbers indicating the distance (in base pairs) from each junction to the start or end of the neighboring gene. Different colored boxes denote distinct regions.

Except for *D*. *densiflorum*, the JLB boundary (the boundary between the LSC and IRb regions) of all other species is located within the *rpl2* gene, approximately 1484 bp from the start site of this gene (Figure 2). The JLA boundary (the boundary between the IRa and LSC regions) of all species is situated on the same side as the trnH gene, with an interval of approximately 75 bp from the trnH gene. The JSB boundary (the boundary between the IRb and SSC regions) is near the right end of the ndhF gene in most species, at a distance of 68–72 bp, whereas this boundary has shifted in *D. densiflorum* due to IR region expansion. The JSA boundary (the boundary between the SSC and IRa regions) is located within the *ycf1* gene; *D. densiflorum* exhibits a fragmented duplication of the *ycf1* gene at this boundary, with sequences duplicated at positions 3639 bp and 1677 bp, and a 27 bp short sequence immediately following the second duplication.

The length of the IR region in the chloroplast genomes of the 12 species ranges from 25,977 bp (*D*. *anthriscifolium*) to 26,565 bp (*Delphinium denudatum*). Among them, the IR region of *D. densiflorum* is 26,558 bp in length, and its SSC region is 16,164 bp, the shortest among the tested *Delphinium* species—further confirming that its IR boundary has undergone significant expansion toward the SSC region.

### 3.3. Sequence Variation Analysis of Delphinium Chloroplast Genomes

**Figure 3:**
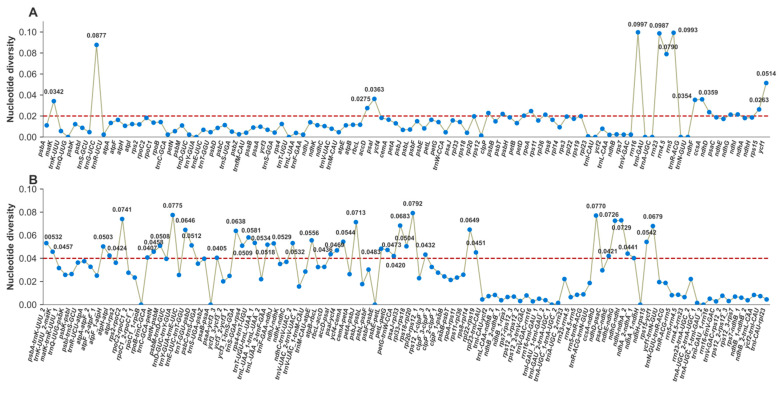
Nucleotide diversity (Pi) of chloroplast genome regions in *Delphinium*. (**A**) Protein-coding genes (CDSs). (**B**) Intergenic spacers (IGSs). The red dashed line indicates the threshold for conserved and hypervariable regions.

Global multiple sequence alignment of *Delphinium* chloroplast genomes revealed significant sequence divergence concentrated in non-coding regions. Four hypervariable regions were identified: *trnK-rbcL*, *psbZ-psaB*, *rps7-ndhE*, and *rpl32-ycf1* (Figure 3).

Nucleotide diversity (Pi) analysis was performed on 87 protein-coding genes (CDSs) and 114 intergenic spacers (IGSs). For protein-coding genes (Figure 3A), Pi values ranged from 0.0013 (*rps12*) to 0.0514 (*ycf1*). Four photosynthetic core genes showed extreme conservation with Pi ≤ 0.01: *rps12* (Pi = 0.0013), *ndhB* (Pi = 0.0026), *psbZ* (Pi = 0.0027), and *psbE* (Pi = 0.0082). In contrast, six genes exhibited elevated divergence (Pi ≥ 0.02): *ycf1* (Pi = 0.0514), *psaI* (Pi = 0.0363), *ccsA* (Pi = 0.0359), *ndhF* (Pi = 0.0354), *matK* (Pi = 0.0342), and *rpoA* (Pi = 0.0247). Notably, ribosomal RNA genes (*rrn16*, *rrn23*, and *rrn5*) displayed the highest diversity among all functional categories (Pi = 0.0987–0.0997).

**Figure 4:**
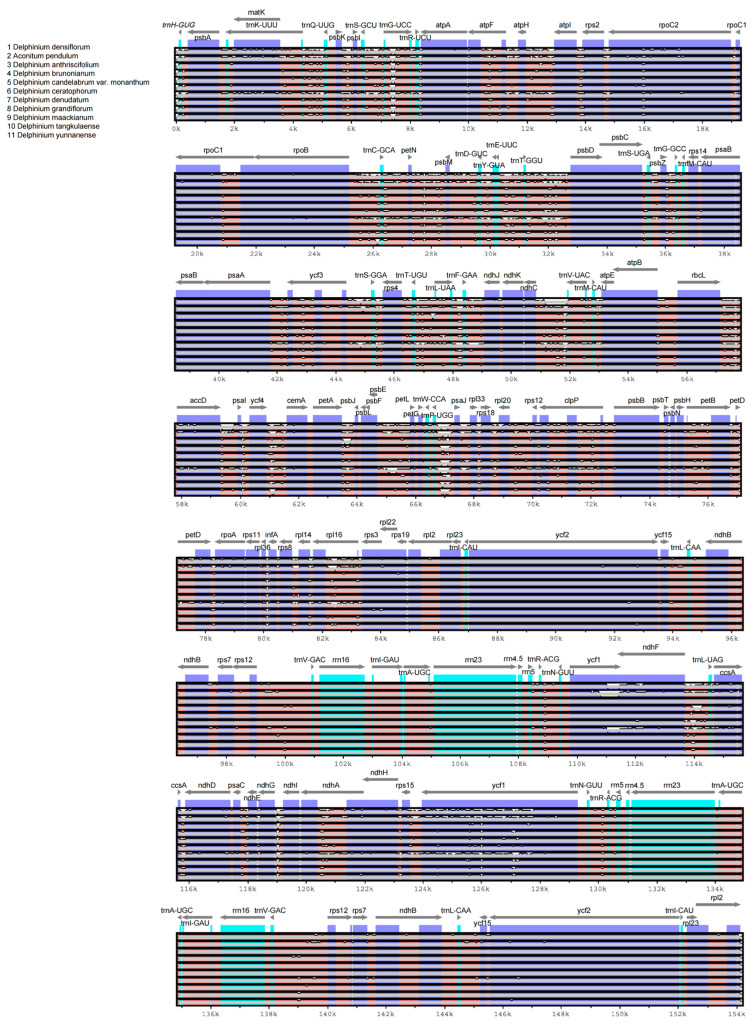
Sequence alignment of chloroplast genomes from *D. densiflorum* and related species using mVISTA. The *x*-axis represents the position in the reference genome (*D. densiflorum*). Purple bars indicate exons; gray arrows show gene orientation and transcription direction. Pink/red areas denote regions of high sequence conservation (≥70% identity), while light blue areas indicate non-coding or less conserved regions. Black vertical lines represent SNPs; green vertical lines indicate indels.

Intergenic spacers (IGSs) showed significantly higher divergence than coding regions (Figure 3B), with Pi ranging from 0.0000 to 0.0792. Eight IGS regions qualified as hypervariable hotspots (Pi ≥ 0.06) and are suitable as DNA barcode candidates: *rps18*-*rpl20* (Pi = 0.0792), *trnD-GUC*-*trnY-GUA* (Pi = 0.0775), *ccsA*-*ndhD* (Pi = 0.0770), *ndhG*-*ndhI* (Pi = 0.0729), *psaJ*-*rpl33* (Pi = 0.0683), *petA*-*psbJ* (Pi = 0.0713), *rps15*-*ycf1* (Pi = 0.0679), and *trnE-UUC*-*trnT-GGU* (Pi = 0.0646). These quantitative data confirm the hypervariable regions identified in the whole-genome alignment (Figure 4). At the whole chloroplast genome level, *D. densiflorum* showed higher similarity to alpine species such as *D. caeruleum* and *D. denudatum*.

To assess the robustness of hypervariable hotspot identification, we performed sensitivity analyses by varying sliding-window parameters and taxon sampling. Using window sizes of 400, 600, and 800 bp (with proportional step sizes of 133, 200, and 267 bp), the six hypervariable CDS (*ycf1*, *psaC*, *rps11*, *rpl22*, *rps18*, and *rps16*) and eight IGS (*rps18*-*rpl20*, *trnD-GUC*-*trnY-GUA*, *ccsA*-*ndhD*, *ndhG*-*ndhI*, *psaJ*-*rpl33*, *petA*-*psbJ*, *rps15*-*ycf1*, and *trnE-UUC-trnT-GGU*) regions remained consistent across all parameter combinations. Similarly, jackknife resampling (iteratively excluding one of the 12 species) recovered the same hotspot regions in >90% of iterations, indicating that the results are robust to single-taxon exclusion.

### 3.4. Results of Codon Usage Bias (RSCU) Analysis

**Figure 5:**
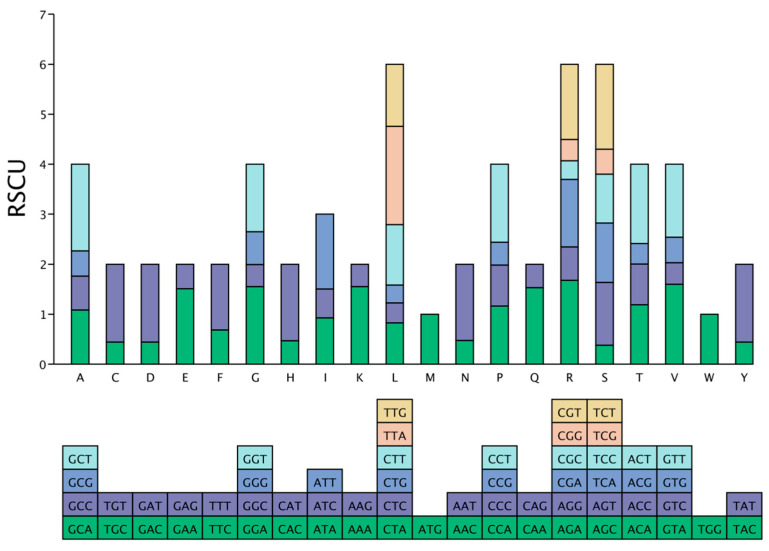
Relative Synonymous Codon Usage (RSCU) Analysis of Various Amino Acids in the Chloroplast Genome of *D. densiflorum*. Note: The upper and lower parts of the bar chart represent the total usage frequency of all synonymous codons and all synonymous codons corresponding to each amino acid, respectively.

Based on RSCU (Relative Synonymous Codon Usage) profile analysis (Figure 5), the number of encodings among synonymous codons for amino acids in the chloroplast genome of *D*. *densiflorum* exhibited differences. Methionine (Met) and Tryptophan (*Trp*) were each encoded by a single codon; nine amino acids—Cysteine (*Cys*), Aspartic acid (*Asp*), Glutamic acid (*Glu*), Phenylalanine (*Phe*), Histidine (*His*), Lysine (*Lys*), Asparagine (*Asn*), Glutamine (*Gln*), and Tyrosine (*Tyr*)—were each encoded by two codons; Isoleucine (*Ile*) was encoded by three codons; five amino acids—Alanine (*Ala*), Glycine (*Gly*), Proline (*Pro*), Threonine (*Thr*), and Valine (*Val*)—were each encoded by four codons; and three amino acids—Leucine (*Leu*), Arginine (*Arg*), and Serine (Ser)—were each encoded by six codons.

Analysis of the 64 codons in the *D. densiflorum* chloroplast genome revealed that 32 synonymous codons had a high usage frequency (RSCU ≥ 1), of which 87.50% (28/32) ended with A/U bases. This indicates that the codons used in the chloroplast genome of *D. densiflorum* show a strong bias toward A/U-ending.

### 3.5. Phylogenetic Relationships

**Figure 6:**
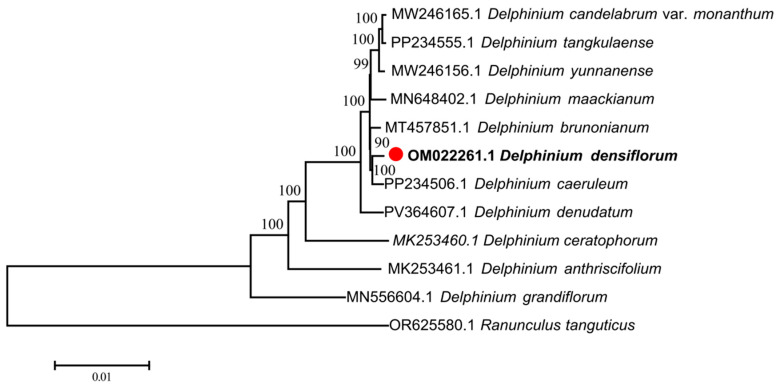
Maximum-likelihood (ML) phylogenetic tree of *D. densiflorum* and its closely related taxa.

The phylogenetic tree (Figure 6) constructed using the maximum likelihood method (ML, GTR model, bootstrap = 1000) indicates that when *R. tanguticus* is designated as the outgroup, *D. densiflorum* is consistently situated within the core group of the genus *Delphinium*. Along with *D. caeruleum* and *D. denudatum*, it forms a monophyletic branch with robust support (BS = 100), categorized as the “Himalaya-Hengduan Rapid Radiating Branch” [31,32]. This branch encompasses six high-altitude species, including *D. brunonianum* and *D. maackianum*. In contrast, *D. grandiflorum*, which exhibits similar morphology, is positioned at the base of the phylogenetic tree and is more closely related to the core group (BS = 100). These findings are consistent with the hypothesis of non-monophyly of the genus *Delphinium* based on the maternal-inherited chloroplast genome. However, we caution that plastid phylogenies reflect only the maternal evolutionary history and may not fully represent the species tree, particularly given the documented hybridization and polyploidization events in this genus [8,9]. Nuclear ribosomal (ITS) or low-copy nuclear gene data are required to further test this phylogenetic hypothesis.

## 4. Discussion

### 4.1. Conservation and Distinctiveness of Chloroplast Genome Architecture

Here we present the first complete chloroplast genome of *D. densiflorum*. It retains the canonical quadripartite circular organization of angiosperms, with a GC content of 38.24% and 131 unique genes—both parameters lying within the range reported for other *Delphinium* species, thereby underscoring the macrostructural conservatism of the genus [33]. Nevertheless, *D. densiflorum* displays a pronounced expansion of the inverted-repeat (IR) region to 26,558 bp, concomitantly reducing the small single-copy (SSC) region to 16,164 bp—the shortest SSC recorded in *Delphinium*. This IR elongation is accompanied by autapomorphic structural changes, including a fragmented duplication of *ycf1* and a truncated *rps19*. Similar IR-boundary dynamics have been documented in other alpine lineages, such as Saxifraga species from the European Alps [34] and Rhododendron species distributed across high-elevation gradients [35]. These convergent patterns are consistent with IR expansion-contraction, potentially reflecting a recurrent genomic response to high-altitude environments, though alternative explanations involving shared ancestral polymorphisms or mutational hotspots at IR-SSC junctions cannot be excluded [36]. However, whether these structural changes constitute direct adaptive mechanisms (e.g., safeguarding photosynthetic stability via gene-dosage effects) or result from neutral evolutionary processes and genetic drift remains to be functionally validated [36].

We acknowledge that IR boundary shifts may arise from neutral mutational processes rather than selection. The IR-SSC junctions are known hotspots for illegitimate recombination and slipped-strand mispairing, which can generate expansion-contraction cycles independent of environmental adaptation [34]. The observed correlation between IR expansion and high-altitude distribution in *Delphinium* could therefore reflect: (i) shared ancestry of alpine lineages rather than convergent adaptation; (ii) genetic drift in small, isolated populations; or (iii) linkage with selected loci elsewhere in the genome. Distinguishing among these hypotheses requires population-level sampling and functional validation.

### 4.2. IR-Boundary Dynamics and Phylogenetic Signal Within the Genus

Comparative mapping of IR/SC junctions across 12 chloroplast genomes reveals pronounced interspecific shifts within *Delphinium*, yet the movement adheres to a “micro-slide” model: the JLA (IRa/LSC) and JLB (LSC/IRb) boundaries remain effectively stationary, whereas JSB (IRb/SSC) and JSA (SSC/IRa) undergo concerted displacement in *D. densiflorum* and its high-elevation allies. This synchronous repositioning constitutes a molecular synapomorphy that recovers the “plateau clade” with 100% bootstrap support [7,37]. While this boundary congruence provides a useful diagnostic trait for distinguishing high-altitude species, it does not necessarily imply adaptive significance; the shared structural pattern may equally reflect shared ancestry or common mutational tendencies at the IR-SSC junctions in this lineage.

### 4.3. Hypervariable Hotspots and DNA-Barcoding Potential

Sliding-window analysis identified four divergence hotspots (Pi ≥ 0.05) within the intergenic spacers *trnK-rbcL*, *psbZ-psaB* and *rpl32-ycf1*, whereas core photosynthetic loci (*psbA*, *rbcL*, *atpB*, etc.) remained extremely conserved (Pi ≤ 0.01), exemplifying a “functionally conserved, sequence-diverged” genomic landscape [37,38]. The single-copy gene *ycf1* exhibited the highest overall variability (Pi = 0.1987) and is increasingly advocated as a universal “super-barcode” because of its length polymorphism and strong phylogenetic resolving power [39]. We detected two tandem repeats within *ycf1* (3.6 kb and 1.7 kb) that can be targeted by specific primers for rapid molecular authentication of *D. densiflorum* against its look-alike congeners [39,40].

### 4.4. Codon-Usage Bias: A Potential Correlation with High-Altitude Environment

Relative synonymous codon usage (RSCU) analysis revealed that 87.5% of preferred codons in *D. densiflorum* are AU-ended. This pattern primarily reflects the overall AT-richness (61.76%) of the chloroplast genome, a characteristic common to many angiosperm plastomes due to mutational biases and weak selection on synonymous sites [33,41]. The biased gene conversion and spontaneous deamination of cytosine to thymine in the single-stranded DNA state during replication contribute to the universal AT mutation bias in chloroplast genomes [42]. The plastid tRNA pool is similarly enriched for A/U anticodons, which may reinforce codon preference through translational selection efficiency [43], although the strength of this selection in plastomes is generally considered weak relative to prokaryotes [41].

In addition to these structural and mutational constraints, the observed AU bias has been hypothesized to confer physiological advantages in cold environments. Under the ‘cold-adaptation hypothesis; A/T-rich sequences may reduce DNA/RNA secondary structure stability, potentially facilitating transcription and translation at low temperatures [43,44]. Consistent with this hypothesis, *D. densiflorum* shows AT enrichment comparable to other Himalayan-Hengduan alpine species [10,23]. However, the low-elevation congener *D. grandiflorum* (distributed at <2000 m) exhibits similarly high AU bias (86.3% AU-ending codons) [24], indicating that this pattern is likely phylogenetically conserved across *Delphinium* rather than exclusively driven by altitude-dependent selection. Furthermore, direct experimental evidence linking codon usage to thermal performance is lacking, and the correlation may equally reflect shared ancestry or genetic drift in isolated alpine populations [45].

Distinguishing between neutral and adaptive explanations for codon usage bias requires heterologous expression experiments or comparative transcriptomics across temperature gradients [43]. We therefore interpret the AU-rich profile of *D. densiflorum* as a candidate pattern for functional validation rather than established evidence of adaptation.

### 4.5. Phylogenetic Placement and Evidence for the Non-Monophyly of Delphinium

The maximum-likelihood tree reconstructed from 69 shared CDS loci provisionally groups *D*. *densiflorum* with *D. caeruleum* and *D. denudatum* in a clade receiving 100% bootstrap support, nested within the “Himalaya-Hengduan rapid-radiation lineage.” In contrast, the morphologically similar, low-elevation *D. grandiflorum* is placed at the base of the tree. While this topology suggests potential paraphyly of *Delphinium* with respect to other genera in Delphinieae, we emphasize that chloroplast genomes represent a single, maternally inherited locus susceptible to introgression and incomplete lineage sorting. The observed conflict between morphology and plastid phylogeny—whereby *D. grandiflorum* is distant from high-elevation congeners despite similar floral morphology—is consistent with either (i) convergent evolution of floral traits or (ii) maternal capture of divergent plastid lineages via hybridization [8,12]. Robust inference of generic monophyly requires concordance between plastid and nuclear phylogenies; until such data are available, we interpret the non-monophyly of *Delphinium* as a provisional hypothesis requiring validation through phylogenomic analyses of nuclear markers [8].

## 5. Conclusions

Based on the research background and hypotheses, this study is the first to decode the chloroplast genome of *D*. *densiflorum*, which not only conforms to the conservative characteristics of the genus *Delphinium* but also exhibits unique variations adaptive to high altitudes [23]. With a length of 154,161 bp, a GC content of 38.24%, and 131 annotated genes (87 CDSs, 8 rRNAs, and 37 tRNAs), its composition is highly consistent with previously reported *Delphinium* species, confirming the structural stability of chloroplast genomes in this genus. Notably, the significant expansion of the IR region (26,558 bp in length) toward the SSC region results in the shortest SSC region (16,164 bp) among the tested *Delphinium* species, accompanied by structural variations at the IR boundaries, specifically partial duplication of *ycf1* and truncation of *rps19* [38]. These findings document the structural differentiation of the chloroplast genome in *D. densiflorum*, consistent with patterns observed in other high-altitude lineages. While we discuss potential correlations with environmental pressures, causal relationships between IR expansion and high-altitude adaptation remain hypothetical and require further functional investigation.

Sequence variation analysis reveals a pattern of “conserved photosynthetic core genes (e.g., *psbA*, *rbcL*, with Pi ≤ 0.01) and divergent auxiliary genes (e.g., *ycf1*, *psaC*, with Pi ≥ 0.05)”, while the strong preference for A/U-ending codons (87.50% of high-frequency codons end with A/U) further illustrates the balanced strategy of *D. densiflorum* in maintaining basic life activities and adapting to the low-temperature high-altitude environment [23,44].

Phylogenetic analysis based on maternally inherited chloroplast CDSs provisionally clarifies the evolutionary position of *D. densiflorum* within the context of plastid evolution. This species clusters with high-altitude species such as *D. caeruleum* and *D. denudatum* into a clade with 100% bootstrap support. However, given that chloroplast genomes track only maternal lineage history and are subject to introgression, we tentatively interpret the distant relationship with *D. grandiflorum* as suggestive of potential paraphyly of *Delphinium* sensu lato, pending validation by biparentally inherited nuclear markers. Future studies incorporating ITSs and low-copy nuclear genes are essential to distinguish between true phylogenetic divergence and maternal lineage sorting or cytoplasmic capture events [8,12]. Consistent with previous molecular systematic studies, this result reveals potential misjudgments in traditional morphological classification due to over-reliance on convergent floral traits and confirms the driving role of the Qinghai-Tibet Plateau uplift and climate fluctuations in the narrow endemic specialization and rapid differentiation of species. Additionally, highly variable genes such as *ycf1* and *rps11*, as well as variable hotspots including *trnK-rbcL* and *rpl32-ycf1*, can be developed into efficient DNA barcodes, providing practical technical support for the accurate identification of *Delphinium* species and the protection and rational utilization of medicinal resources.

## Figures and Tables

**Table 1:** Chloroplast genomes used for phylogenetic tree construction.

No	Family	Subfamily	Species	Genbank ID
1	Ranunculaceae	*Ranunculus*	*Ranunculus tanguticus*	OR625580.1
2	Ranunculaceae	*Delphinioideae*	*Delphinium densiflorum*	OM022261.1
3	Ranunculaceae	*Delphinioideae*	*Delphinium anthriscifolium*	MK253461.1
4	Ranunculaceae	*Delphinioideae*	*Delphinium brunonianum*	MT457851.1
5	Ranunculaceae	*Delphinioideae*	*Delphinium caeruleum*	PP234506.1
6	Ranunculaceae	*Delphinioideae*	*Delphinium candelabrum* var. *monanthum*	MW246165.1
7	Ranunculaceae	*Delphinioideae*	*Delphinium ceratophorum*	MK253460.1
8	Ranunculaceae	*Delphinioideae*	*Delphinium denudatum*	PV364607.1
9	Ranunculaceae	*Delphinioideae*	*Delphinium grandiflorum*	MN556604.1
10	Ranunculaceae	*Delphinioideae*	*Delphinium maackianum*	MN648402.1
11	Ranunculaceae	*Delphinioideae*	*Delphinium tangkulaense*	PP234555.1
12	Ranunculaceae	*Delphinioideae*	*Delphinium yunnanense*	MW246156.1

**Table 2:** Gene composition of the *D. densiflorum* chloroplast genome.

Function	Gene Grouping	Gene Name
Photosynthesisrelated genes	photosystem I	*psaA*, *psaB*, *psaC*, *psaI*, *psaJ*
	photosystem II	*psbA*, *psbB*, *psbC*, *psbD*, *psbE*, *psbF*, *psbH*, *psbI*, *psbJ*, *psbK*, *psbL*, *psbM*, *psbN*, *psbT*, *psbZ*
	cytochrome b/f complex	*petA*, petB*, petD*, *petG*, *petL*, *petN*
	ATP synthesis	*atpA*, *atpB*, *atpE*, atpF*, *atpH*, *atpI*
	NADH dehydrogenase	ndhA*, ndhB*(2), *ndhC*, ndhD*, *ndhE*, *ndhF*, *ndhG*, *ndhH*, *ndhI*, ndhJ*, *ndhK*
	RubisCO large subunit	*rbcL*
Self-replication	RNA polymerase	*rpoA*, *rpoB*, rpoC1*, rpoC2*
	Large ribosomal protein	rpl2**(2), *rpl14*, rpl16*, *rpl20*, *rpl22*, *rpl23*, *rpl33*, *rpl36*
	Small ribosomal protein	*rps2*, rps3*, *rps4*, *rps7(2)*, *rps8*, *rps11*, rps12**(2), *rps14*, *rps15*, rps16*, *rps18*, *rps19*
	Ribosomal RNAs	*rrn16(2)*, *rrn23(2)*, *rrn4.5(2)*, *rrn5(2)*
	Transfer RNAs	trnA-UGC*(2), *trnC-GCA*, *trnD-GUC*, *trnE-UUC*, *trnF-GAA*, *trnG-GCC*, *trnG-UCC*, *trnH-GUG*, trnI-CAU*(2), *trnI-GAU*, trnK-UUU*, *trnL-CAA(2)*, trnL-UAA*, trnL-UAG*, *trnM-CAU*, *trnN-GUU(2)*, *trnP-UGG*, *trnQ-UUG*, *trnR-ACG(2)*, *trnR-UCU*, *trnS-GCU*, *trnS-GGA*, *trnS-UGA*, *trnT-GGU*, *trnT-UGU*, *trnV-GAC(2)*, *trnV-UAC*, *trnW-CCA*, trnY-GUA*, *trnfM-CAU*
Other genes	Caseinolytic protease P	clpP*
	Maturase	*matK*
	Translation initiation factor	*infA*
	Chloroplast envelope membrane protein A	*cemA*
	AcetylCoA carboxylase βsubunit	*accD*
	Ctype cytochrome synthesis factor A	*ccsA*
Hypothetical reading frame	hypothetical reading frame	*#ycf1*, *ycf2(2)*, ycf3*, ycf4**, *ycf15(2)*

Notes: Gene*: Gene with one intron; Gene**: Gene with two introns;# Gene: Pseudo gene; Gene *(2)*: Number of copies of multicopy genes.

## Data Availability

The data presented in this study are openly available in the GenBank of NCBI at https://www.ncbi.nlm.nih.gov (accessed on 28 October 2025) under the accession number OM022261.1.
